# Benign Multicystic Peritoneal Mesothelioma in a Male Patient with Previous Wilms' Tumor: A Case Report and Review of the Literature

**DOI:** 10.1155/2018/4324986

**Published:** 2018-08-01

**Authors:** Gian Luigi Canu, Fabio Medas, Giulio Columbano, Luca Gordini, Luca Saba, Enrico Erdas, Pietro Giorgio Calò

**Affiliations:** ^1^Department of Surgical Sciences, “Policlinico Universitario Duilio Casula”, University of Cagliari, 09042 Monserrato, Italy; ^2^Department of Radiology, “Policlinico Universitario Duilio Casula”, University of Cagliari, 09042 Monserrato, Italy

## Abstract

Benign multicystic peritoneal mesothelioma (BMPM) is a rare condition, more common in females of reproductive age, which arises from the peritoneal mesothelium. A 33-year-old male presented to our unit with abdominal pain and constipation. His past medical history included a previous unilateral nephrectomy for Wilms' tumor and the previous incidental finding of some intra-abdominal cystic formations at the level of the mesentery. After performing a CT scan, an exploratory laparotomy was done and a voluminous cystic mesenteric mass, composed of 3 confluent formations, was observed. Some other similar but significantly smaller lesions were found. An en bloc resection of the mesenteric mass together with the corresponding intestinal loops, an appendicectomy, and some peritoneal biopsies were performed. The postoperative period was complicated by a peritonitis due to dehiscence of the intestinal anastomosis, which required another operation, and a delayed return of normal bowel function, which was resolved through prokinetic therapy. Through histological examination, a BMPM was diagnosed. At 8 months of follow-up, the patient is free of symptoms. BMPM exact etiopathogenesis still remains uncertain. Given his high recurrence rate, a long-term follow-up is recommended.

## 1. Introduction

Benign multicystic peritoneal mesothelioma (BMPM) is an uncommon benign tumor that arises from the peritoneal mesothelial cells. It usually affects females of reproductive age. Its etiopathogenesis is still controversial [1, 2]. The prevailing theory considers this tumor as the result of a persistent inflammatory process involving the peritoneum [3]. However, other possible etiopathogenetic mechanisms have also been suggested [4–11]. BMPM has an extremely low potential of malignant transformation, but it has a very high local recurrence rate after therapy [1, 6, 12–14]. To date, surgery is considered the mainstay of treatment [1, 4, 12, 13, 15].

In this paper, we report a case of BMPM in a male patient submitted to unilateral nephrectomy for a right-sided Wilms' tumor when he was a child.

## 2. Case Report

A 33-year-old Caucasian male presented to our surgical unit after 3 days of progressively worsening abdominal pain. The patient also reported the onset of constipation for some days, but he denied any associated nausea or vomiting.

His past medical history included a right-sided Wilms' tumor treated with unilateral nephrectomy when he was 2 years old. Moreover, during the follow-up for this pediatric tumor, some progressively growing intra-abdominal cystic formations, localized at the level of the mesentery, have been incidentally identified.

Physical examination revealed a hypogastric mass and diffuse abdominal tenderness without abdominal distension.

**Figure 1:**
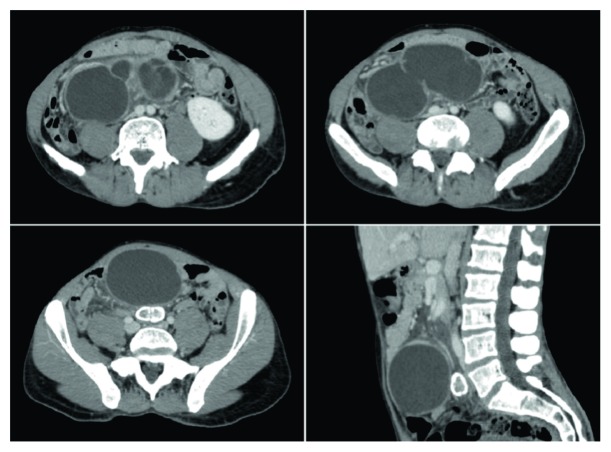
BMPM on CT scan. Contrast-enhanced CT scan shows the presence of 3 contiguous, communicating, and thin-walled cystic formations located at the level of the mesentery. The mass with larger dimensions is the most ventral one, and it is localized in contiguity with the abdominal wall, in the subumbilical region.

The patient underwent an abdominal CT scan (Figure 1) which showed the presence of 3 contiguous and communicating cystic formations located at the level of the mesentery that altogether had a major axis equal to 115 mm. Among these, the mass with larger dimensions (major axis equal to 87 mm) was the most ventral one and it was localized in contiguity with the abdominal wall, in the subumbilical region. Moreover, further intra-abdominal lesions similar to the previous ones, but significantly smaller in size, were described.

**Figure 2:**
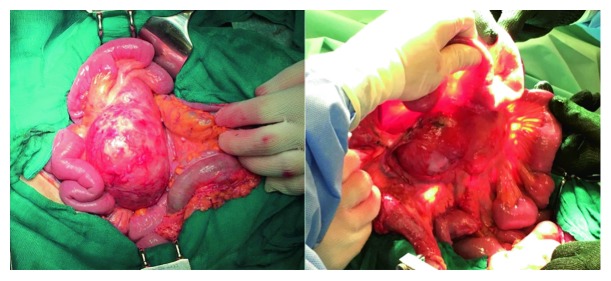
Intraoperative images of BMPM. Voluminous cystic mass incorporated in the mesentery of the last ileal loops detected during the operation.

Due to worsening symptoms, an exploratory laparotomy was performed and a voluminous cystic mass, composed of 3 confluent formations, with a major axis equal to about 10 cm, and incorporated in the mesentery of the last ileal loops, was observed (Figure 2). Some other similar but significantly smaller lesions were found, in particular in the pelvic cavity and in correspondence of the cecal appendix. Thus, an en bloc resection of the voluminous mesenteric formation together with the corresponding intestinal loops with ileoileal anastomosis, an appendicectomy, and some peritoneal biopsies were performed. The whole procedure was hampered by the presence of scar adhesions due to the previous nephrectomy.

The postoperative period was complicated, on the 8th postoperative day, by a circumscribed peritonitis due to dehiscence of the ileoileal anastomosis. The patient was therefore submitted to another operation. On this occasion, a resection of the previous anastomosis and an ileocecal resection with ileo ascending colon anastomosis were performed.

Moreover, the postoperative period was complicated by a delayed return of normal bowel function which was resolved through prokinetic therapy with levosulpiride and neostigmine methylsulfate. The patient was finally discharged 33 days after the first operation in good condition.

Through histological examination a benign multicystic peritoneal mesothelioma was diagnosed.

At 8 months of follow-up, the patient is free of symptoms.

## 3. Discussion

BMPM was first described in 1928 by Plaut, who incidentally observed some pelvic cystic lesions during surgery for uterine leiomyoma [16], but its mesothelial nature was identified in 1979 by Mennemeyer and Smith [17]. To date, less than 200 cases have been reported in literature [1, 2].

This tumor usually affects females of reproductive age, and it is very rare in males [1, 2, 13, 15]. Our experience is particular because our patient is a young man.

Its etiopathogenesis is still debated [1, 2]. The prevailing theory considers BMPM as the result of a persistent inflammatory status involving the peritoneum because of its association with endometriosis, pelvic inflammatory disease, previous abdominal surgery, and recurrent peritonitis episodes associated with peritoneal dialysis and familial Mediterranean fever [3, 18, 19]. According to this hypothesis, a persistent inflammatory process would result in a reactive hyperplastic and dysplastic transformation of mesothelial cells. Some authors have instead suggested a more primitive neoplastic origin without a strict association with an inflammatory insult [4, 5]. This theory is based on a slow but progressive growth of the lesions, the tendency to recur after treatment, and the possibility, even if extremely rare, of malignant transformation. Moreover, a hormonal hypothesis has also been proposed [6, 7]. According to this theory, the development and progression of BMPM would be tightly linked to its sensitivity to sex hormones. This hypothesis is supported by the evidence of higher incidence in females of reproductive age and the responsiveness of this tumor, in some experiences, to some endocrine drugs, as tamoxifen, and gonadotropin-releasing hormone analogs. Some authors have also postulated that the sensitivity to peripheral estrogen sources can explain the high recurrence rate of this tumor after treatment. Finally, some cases of BMPM associated with congenital anomalies have been described [8–11]. These cases occurred in an 11-month-old male with congenital cystic adenomatoid malformation of the right lung [8] and in 4 patients with congenital renal anomalies (a child with contralateral renal agenesis, a teenage female with an extrarenal pelvis kidney, a teenage female with a horseshoe kidney, and a young male with ipsilateral renal agenesis) [9–11]. Thus, a possible developmental origin of BMPM has been postulated, with pathogenesis attributed to developmental abnormalities of the peritoneum [9, 10]. We instead describe a case of BMPM in a patient submitted to unilateral nephrectomy for a right-sided Wilms' tumor during childhood. This pediatric renal tumor (also called Wilms tumor or nephroblastoma) can be associated with numerous congenital anomalies which can occur as isolated entities or within well-defined syndromes (Beckwith-Wiedemann, Denys-Drash, Perlman, Simpson-Golabi-Behmel, and WAGR syndromes) [20–27]. Many of these congenital disorders involve the genitourinary system, including renal ectopia, horseshoe kidney, renal hypoplasia, ureteral duplication, cryptorchidism, hypospadias, and male pseudohermaphroditism. Our patient does not present any congenital malformation, and ultimately, in our case, the onset of BMPM seems to be the effect of the previous abdominal operation for Wilms' tumor.

**Figure 3:**
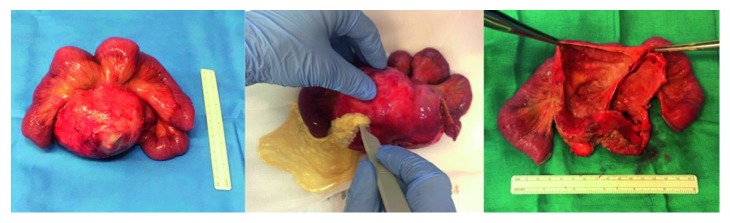
Surgical specimen of BMPM. Voluminous cystic formation, composed of 3 confluent masses, with a major axis equal to about 10 cm and filled with straw-coloured fluid observed after resection.

BMPM has a predilection for the pelvic peritoneum [15, 28, 29]. Multiple cysts forming a confluent mass are usually found, although isolated cystic lesions have also been reported [3, 14]. Cystic lesions can range from a few millimetres to more than 20 cm in size and are often filled with serous fluid which can be straw-coloured or clear [3, 6]. In our case, a voluminous cystic mass, composed of 3 confluent formations, with a major axis equal to about 10 cm, incorporated in the mesentery of the last ileal loops and filled with straw-coloured fluid, was found (Figures 2 and 3).

BMPM is often discovered incidentally; in fact, as in our case, the majority of patients are asymptomatic until this tumor is quite large to cause a mass effect on other organs [1–3, 12, 15]. Clinical manifestations can include abdominal and/or pelvic pain, early satiety, abdominal fullness, unintentional weight gain, changes in bowel habits, and intestinal obstruction.

Physical examination can reveal abdominal distension, abdominal tenderness, and one or more palpable abdominal and/or pelvic masses [2, 15]. Our patient presented with a hypogastric mass and diffuse abdominal tenderness without abdominal distension.

Preoperative diagnosis is challenging [28]. There are some benign and malignant disorders that can simulate BMPM, such as abdominal cystic lymphangioma, pseudomyxoma peritonei, cystic adenomatoid tumor, endometriosis, malignant peritoneal mesothelioma, and secondary tumors involving the peritoneum [2, 28–30].

Ultrasound (US) and, above all, CT scan are useful for diagnosis. In addition, MRI can also be employed [28, 29, 31, 32]. US can demonstrate multiseptated anechoic cystic formations while CT scan usually shows, as in our case (Figure 1), low-density, thin-walled, multiloculated, multicystic masses. CT scan also provides careful information about the location and extent of the lesions. However, US and CT scan do not differentiate BMPM from other similar cystic formations. MRI can instead confirm the peritoneal origin of the lesions and differentiate the cystic content. On MRI, typical findings are low signal on T1WI and high signal on T2WI. Moreover, the cystic walls demonstrate mild enhancement following Gadolinium administration.

Definitive diagnosis requires histological examination, and if any doubts remain, positive immunohistochemical staining of the mesothelial cells for calretinin confirms the diagnosis [15, 28–30, 33].

To date, the main therapy is surgery [1, 4, 12, 13, 15]; however, the ideal treatment continues to be highly debated. Beyond surgery, multiple additional therapies have been tested, such as hormonal therapy with antiestrogen agents or gonadotropin agonists, sclerotherapy with tetracycline, potassium-titanyl-phosphate laser, and hyperthermic intraperitoneal chemotherapy (HIPEC) [2, 6, 7, 34]. In this last case, the most used combination of drugs is composed of cisplatin and doxorubicin [35]. However, all these therapies have had controversial results. We performed surgery without any additional treatment.

Prognosis appears to be very good [1, 2]. To date, only one death has been reported: a 14-year-old patient who died 12 years after diagnosis due to refusal of surgery [36].

BMPM has an extremely low potential of malignant transformation. In literature, there are only 2 cases of malignant transformation [5, 37]. However, this tumor has a very high local recurrence rate after treatment, equal to 41%–50% and occurring from 2 to 36 years after the initial presentation [1, 6, 12–14]. The recurrence rate of BMPM remains high, equal to about 33% in men and 50% in women, even by using the hyperthermic intraperitoneal chemotherapy (HIPEC) in combination with surgery [2]. For this reason, a long-term follow-up after treatment is recommended [1, 4]. Some authors believe that follow-up imaging should be performed; however, given the rarity of this tumor, no established guidelines still exist [15]. At 8 months of follow-up, our patient is free of symptoms.

In conclusion, a new rare case of benign multicystic peritoneal mesothelioma, uncommonly occurred in a male patient, has just been described. BMPM exact etiopathogenesis still remains unclear. The most probable theory, as also suggested by our experience, seems to be the one which considers this tumor as the result of a persistent inflammatory status involving the peritoneum, as in case of previous abdominal operations. To date, despite the various combinations of therapies which have been tested, the local recurrence rate remains high. Thus, a long-term follow-up after treatment is highly recommended.
